# Detection of capripoxvirus DNA using a novel loop-mediated isothermal amplification assay

**DOI:** 10.1186/1746-6148-9-90

**Published:** 2013-05-01

**Authors:** Lee Murray, Lorraine Edwards, Eeva SM Tuppurainen, Katarzyna Bachanek-Bankowska, Chris AL Oura, Valerie Mioulet, Donald P King

**Affiliations:** 1 The Pirbright Institute, Ash Road, Pirbright, Surrey GU24 0NF, UK; 2 Now at: Faculty of Medical Sciences, University of the West Indies, St. Augustine, Trinidad and Tobago

**Keywords:** Capripoxvirus, Diagnostics, Isothermal amplification, Field tests

## Abstract

**Background:**

*Sheep poxvirus* (SPPV), *Goat poxvirus* (GTPV) and *Lumpy skin disease virus* (LSDV) are the most serious poxviruses of ruminants. They are double stranded DNA viruses of the genus *Capripoxvirus*, (subfamily *Chordopoxvirinae*) within the family *Poxviridae*. The aim of this study was to develop a Loop-mediated isothermal AMPlification (LAMP) assay for the detection of Capripoxvirus (CaPV) DNA.

**Results:**

A single LAMP assay targeting a conserved region of the CaPV P32 gene was selected from 3 pilot LAMP assays and optimised by adding loop primers to accelerate the reaction time. This LAMP assay successfully detected DNA prepared from representative CaPV isolates (SPPV, GTPV and LSDV), and did not cross-react with DNA extracted from other mammalian poxviruses. The analytical sensitivity of the LAMP assay was determined to be at least 163 DNA copies/μl which is equivalent to the performance reported for diagnostic real-time PCR currently used for the detection of CaPV. LAMP reactions were monitored with an intercalating dye using a real-time PCR machine, or by agarose-gel electrophoresis. Furthermore, dual labelled LAMP products (generated using internal LAMP primers that were conjugated with either biotin or fluorescein) could be readily visualised using a lateral-flow device.

**Conclusions:**

This study provides a simple and rapid approach to detect CaPV DNA that may have utility for use in the field, or in non-specialised laboratories where expensive equipment is not available.

## Background

Sheeppox virus (SPPV), goatpox virus (GTPV) and lumpy skin disease virus (LSDV) cause serious pox diseases of domesticated ruminants [1]. They are large, complex, double-stranded DNA viruses of the genus *Capripoxvirus*, subfamily *Chordopoxvirinae*, family *Poxviridae*[2]. SPPV and GTPV are restricted to much of Asia, the Middle East and North Africa, although clinical cases of sheep pox have also been detected in Europe [3]. LSDV occurs across Africa and in recent years the virus has also been found in several countries of the Middle East [4]. The World Organization for Animal Health (OIE) classifies capripoxviruses (CaPVs) as notifiable diseases, whilst they are also considered as potential bioterrorism agents due to the economic impact of any large scale outbreak [5].

Molecular diagnostic tests play an important role in monitoring the spread of these viruses and controlling outbreaks in susceptible livestock. Agarose-gel based polymerase chain reaction (PCR) assays [6-10], or more recently developed real-time PCR assays [5,11,12] are rapid and highly sensitive tests widely used in diagnostic laboratories. Real-time PCR assays can also be used to differentiate LSDV, SPPV and GTPV from each other [13]. However, poorly equipped and field laboratories face difficulties accessing these molecular techniques that are reliant upon expensive and relatively fragile equipment. A new group of nucleic acid detection assays that exploit isothermal amplification mechanisms have been developed as potential diagnostic tools for use in either the field or low cost laboratory settings. These assay formats include loop-mediated isothermal amplification (LAMP) [14] which is a DNA-dependent amplification method that uses a combination of four to six primers targeting six to eight genomic regions, whilst utilising the activity of a strand displacing DNA polymerase. The isothermal nature of LAMP potentially allows reactions to be performed simply in a water bath or using a heat pack, usually at a temperature between 60-65°C. These factors make LAMP an ideal candidate for use as the basis of an inexpensive test for use in the field. LAMP assays have been developed for the detection of a wide range of viruses, parasites and bacteria including foot-and-mouth disease virus [15], human immunodeficiency virus (HIV-1) [16], malaria protozoan *Plasmodium* in blood samples [17] and *Escherichia coli*[18]. The aim of this study was to develop and evaluate a LAMP assay for the detection of CaPV DNA and to evaluate this assay using different detection formats that might be suitable for use in simple laboratories.

## Results

### Optimisation of the Capripoxvirus LAMP assay

**Figure 1:**
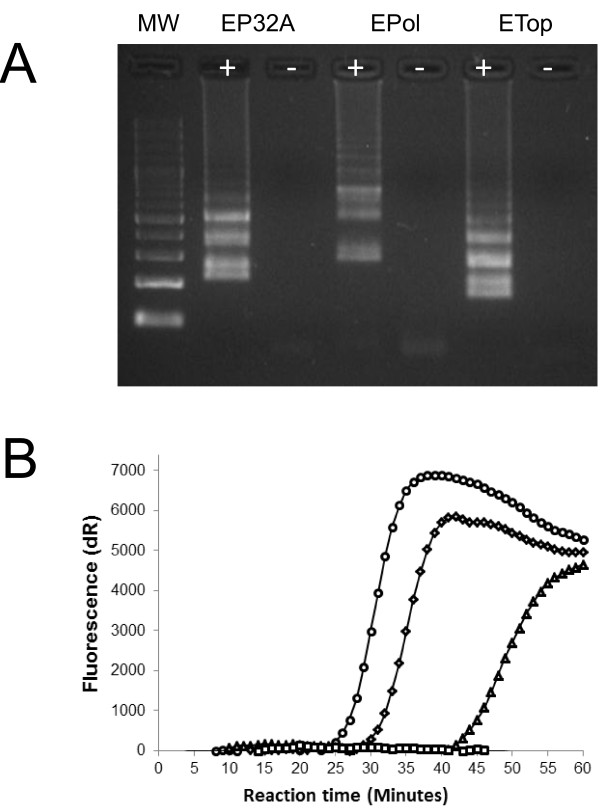
**Evaluation of candidate LAMP assays. A**: Agarose gel (2%) showing the characteristic laddering pattern generated by LAMP for the detection of GTPV isolate Vietnam Ninh Tuan 05 by three different LAMP primer sets: EP32A (P32 gene); Epol (RNA polymerase RPO30 subunit) and ETop (DNA topoisomerase I). Results for negative controls (−) are shown. MW: molecular weight ladder (100 bp). **B**: Corresponding increase in fluorescence generated for EP32A (○: circle); Epol (△: triangle) and ETop (◊: diamond) LAMP assays using a real-time PCR machine. Representative signal for a negative control (□) well is shown.

**Figure 2:**
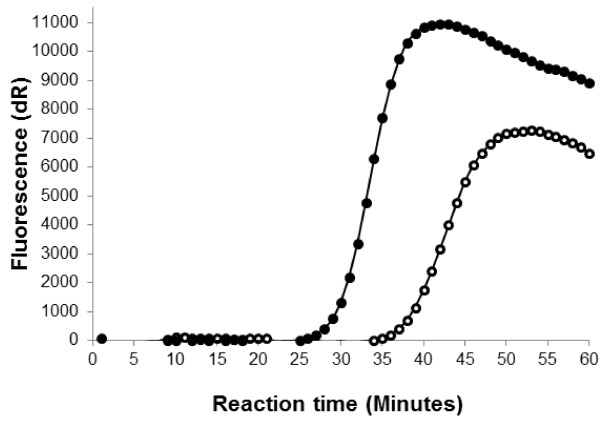
**Acceleration of the CaPV P32 LAMP assay using loop primers.** The results of this representative experiment show the overall speed of detection of DNA from a GTPV isolate (Vietnam Ninh Tuan 05) was decreased by ± 9 minutes using loop primers (●: closed circle) compared with a parallel LAMP reaction without loop primers (○: open circle).

In initial experiments, all three candidate LAMP assays that targeted the RNA polymerase subunit RPO30, DNA topoisomerase I and the P32 regions generated characteristic laddering patterns after agarose-gel electrophoresis (Figure 1A) and an increase in fluorescence in a real-time PCR machine (Figure 1B). However, these experiments indicated that the P32 LAMP assay was the most rapid and reliable assay, and therefore this assay was selected for further optimisation and evaluation. The optimum reagent concentrations in the LAMP reactions mixes were 1× Thermopol buffer (New England Biolabs, Hitchin, UK), 3 μM internal primers, 0.6 μM external primers, 1 mM MgSO_4_ (New England Biolabs, Hitchin, UK), 0.3 mM dNTPs (Sigma-Aldrich, Dorset, UK), 1 M betaine (Sigma-Aldrich, Dorset, UK), 16 U of Bst DNA polymerase (Large fragment: (New England Biolabs, Hitchin, UK), along with the addition of 2 μl of target DNA. Using the P32 primer set, loop primers were added to the reaction at an optimal concentration of 4 μM. These loop primers decreased the reaction time by nine minutes for a GTPV isolate (Vietnam Ninh Tuan 05; Figure 2). Restriction enzyme digests were performed on the P32 LAMP amplification products to confirm that the correct region of the CaPV genome had been amplified. A restriction endonuclease (*BsrG I*) was chosen to cut specifically a single site within the target region of the P32 LAMP assay. After incubation of 5 μl LAMP product with *BsrG I* at 37°C for 75 min, the digested products were visualised on a 4.0% agarose gel and could be differentiated from the characteristic laddering pattern of the LAMP reaction products confirming the sequence specificity of the DNA amplicons (data not shown).

### Diagnostic performance of the LAMP assay

**Figure 3:**
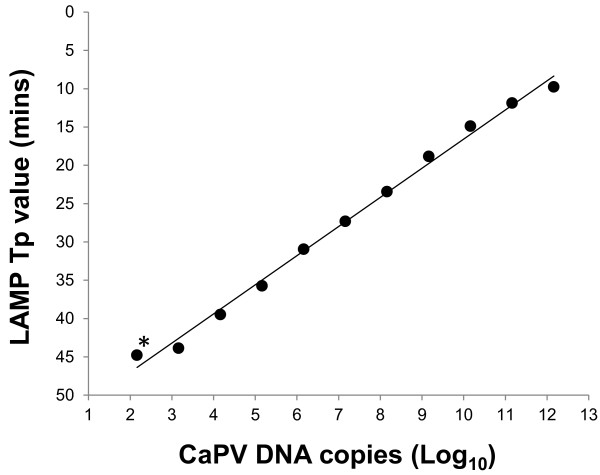
**Analytical sensitivity of the CaPV LAMP assay.** Results (time to positive: T_P_) shown are mean of duplicate samples of DNA generated from a log_10_ dilution series of the GTPV isolate (Vietnam Ninh Tuan 05). For the weakest dilution, only one of the duplicates was positive using the LAMP assay*.

The diagnostic sensitivity of the CaPV LAMP assay was evaluated using 47 CaPV strains that were tested in parallel using the real-time PCR assay used by the OIE reference laboratory at the Pirbright Institute. In comparison to the PCR assay, all of the 47 strains generated positive LAMP results (Table 1). Two isolates (GTPV Pakistan and SPPV Mongolia) initially yielded negative results with the LAMP assay, but gave positive results when retested with fresh nucleic acid preparations. All of the nucleic acid samples prepared from CaPV-negative blood and skin generated negative results in the LAMP assay. In addition, none of the six related poxviruses tested positive, indicating a high specificity of the LAMP assay (Table 1). Using the dilution series of GTPV isolate (Vietnam Ninh Tuan 05), the dynamic range of the CaPV LAMP assay targeting the P32 region was at least 10 log_10_ dilutions and the weakest dilution that could be detected was 163 copies/μl for GTPV isolate (Vietnam Ninh Tuan 05: Figure 3).

**Table 1:** Diagnostic sensitivity and specificity of the CaPV LAMP assay as tested against representative strains of CaPV

**Virus**	**Isolate name**	**LAMP result (T**_**p**_**)**^**a**^	**PCR result (C**_**t**_**)**^**b**^
GTPV	Yemen	31.89	20.98
GTPV	Turkey	23.89	20.77
GTPV	Saudi Arabia	33.62	17.67
GTPV	Oman	27.17	22.96
GTPV	Mongolia	22.25	20.27
GTPV	India	22.65	19.44
GTPV	Held	29.81	24.01
GTPV	Gorgan (Iran)	34.34	21.97
GTPV	Ghana	38.10	26.05
GTPV	China	44.54	36.42
GTPV	Bangladesh	32.27	21.74
GTPV	Abu Ghraib (Iraq)	27.27	17.29
GTPV	Kano (Nigeria)	23.57	21.47
GTPV	Morocco	33.59	21.35
GTPV	Pakistan	44.96	40.28
GTPV	Vietnam	28.96	22.95
SP/GTPV	Kenya	29.62	18.22
SP/GTPV	Saudi Arabia	37.84	21.39
SP/GTPV	Nigeria	42.30	27.98
SP/GTPV	Yemen	29.56	22.75
SP/GTPV	KS-1	36.77	22.60
SPPV	Romania	37.14	24.78
SPPV	Abu Ghraib (Iraq)	34.91	21.84
SPPV	Isiolo (Kenya)	27.66	23.84
SPPV	Kedong (China)	24.83	21.75
SPPV	Mongolia	29.54	17.65
SPPV	Nigeria	36.19	29.03
SPPV	Oman	26.91	19.63
SPPV	Pakistan	38.79	22.60
SPPV	Senegal	40.02	21.71
SPPV	Cyprus	58.85	34.38
SPPV	India	41.68	23.52
SPPV	Sudan	32.35	21.03
SPPV Vaccine Strain	Saudi Arabia	38.05	22.89
SPPV Vaccine Strain	Stavropol (Russia)	37.61	20.79
SPPV Vaccine Strain	France	38.33	21.62
SPPV Vaccine Strain	Pendik (Turkey)	36.38	22.27
LSDV	Israel	27.75	20.96
LSDV	Nigeria	30.16	21.82
LSDV	Uganda	23.73	18.69
LSDV	Senegal	29.22	23.26
LSDV	Cameroon	31.49	21.50
LSDV	Egypt 93	31.41	23.37
LSDV	Egypt 2006	32.14	26.09
LSDV	Neethling	26.98	19.59
LSDV	Oman	32.19	32.19
LSDV	Ghana	28.55	22.63
n/a	Negative Cattle Blood (n = 14)	Negative (n = 14)	Negative (n = 14)
n/a	Negative Sheep Blood (n = 8)	Negative (n = 8)	Negative (n = 8)
n/a	Negative Cattle Skin (n = 16)	Negative (n = 16)	Negative (n = 16)
Buffalopox virus	n/a	Negative	Not Tested
Camelpox virus	n/a	Negative	Not Tested
Cheetah poxvirus	n/a	Negative	Not Tested
Cowpox virus	n/a	Negative	Not Tested
Orf virus	n/a	Negative	Not Tested
Swine poxvirus	n/a	Negative	Not Tested

^a^ T_*p*_ = Time to positive (minutes).

^b^ C_*t*_ = threshold cycle value.

### Simple approaches to detect LAMP products

**Figure 4:**
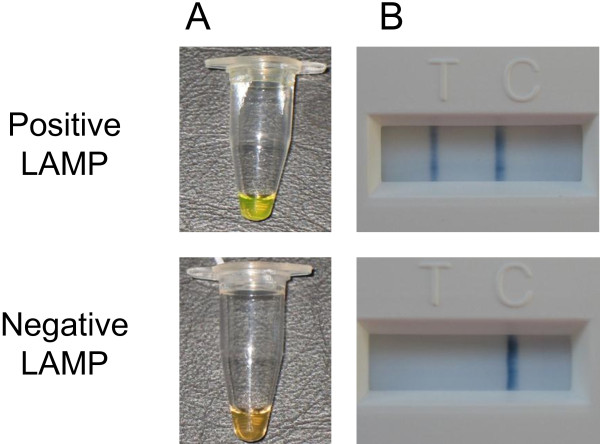
**Successful differentiation of positive and negative sample using simple detection approaches. A**: shows the colour change produced by a representative positive sample (Vietnam Ninh Tuan 05) in comparison to a negative sample after addition of Picogreen. **B**: shows the detection of dual labelled CaPV LAMP products using lateral-flow devices. A band at the “T-line” indicates that the LAMP reaction is positive.

Two alternative simple approaches for detecting the LAMP products were assessed. These methods offer approaches that might be used to deploy the LAMP assay into the field or into simple laboratory settings. A LFD that detects dual labelled nucleic acid products (Forsite Diagnostics, York, UK) was evaluated by adding 2 μl of amplified LAMP product to 198 μl of the dilution buffer (Forsite Diagnostics, York, UK). Positive samples could be clearly discriminated from negative samples using the modified internal LAMP oligonucleotides containing biotin and FITC (Figure 4). In addition, 5 μl of intercalating dye (Picogreen, Molecular Probes, Invitrogen, Paisley, UK) also clearly differentiated between negative and positive samples after amplification by the LAMP assay.

## Discussion

This report describes the development of a new LAMP assay to detect representative members of the *Capripoxvirus* genus that infect domesticated ruminants. A fast, affordable and reliable test for the detection of CaPVs is urgently needed in endemic countries to provide local diagnostic capacity to monitor the spread of these viruses. LAMP provides an alternative to PCR-based assays and due to its isothermal nature may be more suitable for the detection of CaPV in front-line or mobile laboratories in developing countries. Previously, amplification of monkeypox virus by LAMP has been reported [19], along with development of other LAMP assays to detect the causative agents of infectious viral diseases of sheep, pigs, goats and cattle [15,20-22]. More recently, LAMP assays targeting conserved genes encoding the poly(A) polymerase small subunit (VP39) and P32 regions of CaPV genome using hydroxynapthol blue as an indicator dye have been described [23]. In this study, LAMP assays targeting the P32, RNA polymerase subunit RPO30 and DNA topoisomerase I were designed and assessed in initial experiments. The performance of one of these assays (P32) was evaluated using a panel of DNA prepared from different CaPVs.

Using DNA generated from a GTPV isolate, the detection limit of the CaPV LAMP assay was found to be at least 163/μl copies, which is comparable to the diagnostic real-time PCR assay detection limit of at least 125 copies/μl [12]. This corroborates the findings of other livestock LAMP assays which have shown equivalent analytical sensitivity between LAMP assays and PCR assays [15,22,23]. Of the eight regions targeted by the LAMP oligonucleotides, four (FLoop, BLoop, B2 and B3) were completely conserved across all of the 32 sequences in the alignment, while the remaining sites had a small number of nucleotide substitutions for some of the CaPV sequences (consisting of 3, 4, 1, and 3 substitutions for F3, F2, F1C and B1C oligonucleotides, respectively). In view of this sequence heterogeneity among CaPVs, it is likely that the detection limit for the LAMP assay will be different across the CaPV isolates where sequences mismatches are present in critical regions targeted by the oligonucleotides. The CaPV LAMP assay was able to detect DNA from representative strains of SPPV, GTPV and LSDV (Table 1), although sequence data was not available in this current study to assess the influence of nucleotide mismatches upon the signal generated in the assay.

In comparison to real-time PCR, this LAMP assay is a rapid method to detect CaPV DNA. Although Table 1 directly compares the results from these two tests, it is important to remember that the individual PCR cycles are longer (comprising the ramp times between different temperatures of the component PCR steps) than the T_p_ “cycles” used to report the isothermal LAMP assay which directly reports the time (in minutes) for a positive result to be generated. For a real-time CaPV PCR assay, the overall length of the test is approximately 75 minutes, whilst for a gel-based PCR it is over 120 minutes. In this study, the LAMP reactions were incubated for 60 minutes: a positive result was defined as a positive signal within 60 minutes, while a negative result was designated for samples that did not yield a detectable LAMP product after this time. Depending upon the detection format used future work with the LAMP assays will be required to define an appropriate assay time to allow positive and negative samples to be clearly differentiated.

The successful application of the CaPV LAMP assay onto a LFD with the use of ligand-labelled internal primers provides a potential simple-to-use tool for field based detection of the virus and is a less ambiguous method than the hydroxynapthol blue indicator method that has been previously described for CaPV [23]. An important consideration that will need to be accommodated in the development of robust LAMP tests for routine use is to minimise the potential for cross-contamination between samples. Existing simple detection methods (such as LFD or intercalating dye approaches) require opening of reaction tubes after amplification which in their current format may not be ideal for routine use. However, practical solutions to this problem may be achieved using new diagnostic platforms for LAMP that are currently under development. In order to be able to deploy this assay into a simple field format, a simple nucleic acid extraction procedure also needs to be developed, preferably not requiring any form of lengthy kit-based extraction process. Previous studies have noted that LAMP is less likely to be affected by any possible contaminants from the DNA isolation process and will still effectively amplify nucleic acid derived from relatively simple methods of extraction [24]. The most common of these crude methods of extraction appears to be a heat treatment approach performed at 95°C for 10 minutes on blood samples. This has previously led to the detection of both viruses and protozoa [16,17]. Research into these procedures would greatly reduce the number of steps needed prior to the use of LAMP in conjunction with a LFD.

## Conclusions

This new isothermal assay for the detection of CaPV DNA, provides a rapid and simple approach that can be deployed to support improved reporting systems to combat the spread of these important livestock diseases.

## Methods

### Primer design

**Figure 5:**
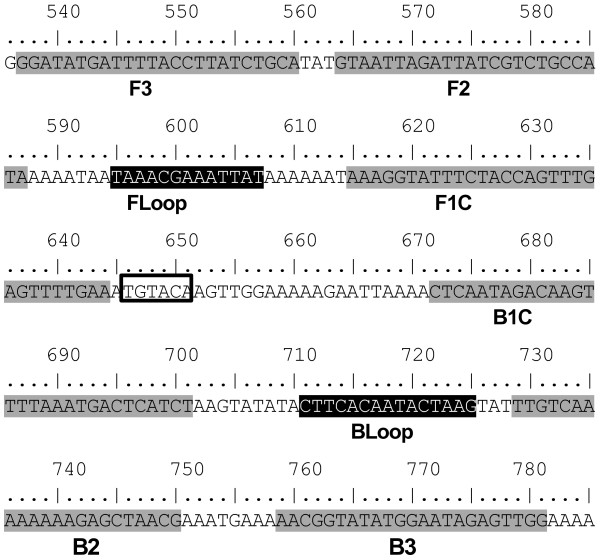
**Schematic showing the location of LAMP oligonucleotide recognition sites within the P32 gene of CaPVs.** Internal and external primer regions are shaded in grey, whilst loop primer regions are highlighted in black. The numbers refer to the position of the nucleotides within the P32 gene for the GTPV sequence (Vietnam Ninh Tuan 2005; GenBank accession number EU625263). The *BsrG I* restriction enzyme recognition site (TGTACA: boxed text) used to confirm amplicon specificity is located between positions 646–651.

Alignments (BioEdit; [25]) containing available CaPV sequences (GenBank) were prepared for the RNA polymerase subunit RPO30, DNA topoisomerase I and the P32 regions. Oligonucleotide primers (Table 2) for LAMP assays targeting these three regions were designed using Primer Explorer V4 software (Fujitsu System Solutions Ltd., Tokyo, Japan). The exact nucleotide positions of the P32 primer set can be seen in Figure 5. In-silico analysis was also undertaken using alignments of homologous regions of other related pox viruses (members of the subfamily *Chordopoxvirinae*, including vaccinia virus and contagious pustular dermatitis (Orf) virus) to ensure that the regions targeted by these different primers were likely to be specific.

**Table 2:** Oligonucleotide primers designed for LAMP assays targeting the P32, RNA polymerase subunit RPO30 and DNA topoisomerase I

**Target Region**	**Primer**	**Length**	**Position**	**Sequence (5′ – 3′)**
**P32**	FIP	58	F1c 610-639	TTCAAAACTCAAACTGGTAGAAATACCTTT-
			F2 559-582	-GTAATTAGATTATCGTCTGCCATA
	BIP	56	B1c 667-696	CTCAATAGACAAGTTTTAAATGACTCATCT-
			B2 724-745	-CGTTAGCTCTTTTTTTTGACAA
	F3	24	532-555	GGATATGATTTTACCTTATCTGCA
	B3	23	754-776	CCAACTCTATTCCATATACCGTT
	FLoop	13	590-602	ATAATTTCGTTTA
	BLoop	15	706-720	CTTCACAATACTAAG
**RNA Polymerase**	FIP	59	F1c 300-331	CTGTTCCATTTGTAGTACGTATAAGATTACAT-
**Subunit RPO30**			F2 244-266	-ATTATCGTATAGAAACAAGCCTT
	BIP	55	B1c 437-468	ATGTTTTAGATAAAAAGTATAACCTCCCATGC-
			B2 487-505	-TCATGACGGGAATAGTGTT
	F3	21	195-215	TGAACCAAGAAACAACATAGG
	B3	18	507-524	TCTGCTGCTCTTGTTTGT
**DNA**	FIP	54	F1c 95-117	GTGGGGCGGTATTCTAATCTTTT-
**Topoisomerase I**			F2 56-81	-CTCTTGTATCATTTGATAATCCTACT
	BIP	52	B1c 135-159	TTACGAACAAACGTATGAACAGTCA-
			B2 178-199	-TACCTTTCGAATCTGAACCAAC
	F3	23	21-43	CGATGGAAAACTTTTTACGGATA
	B3	24	203-226	GCAGTTTTCCATAAAAATATTGCC

### Optimisation of LAMP assay

Nucleic acid prepared from two GTPV isolates (Bangladesh 1986 and Vietnam Ninh Tuan 2005) was used in initial experiments to evaluate the LAMP assays. This material was prepared from cell culture of the viral strains in lamb testis primary cells using Dulbecco’s Modified Eagle’s Medium (Gibco® by Life Technologies™, Paisley, UK) containing 5% foetal calf serum (PAA Laboratories, Yeovil, UK). Infected cells (corresponding to a viral titre that was typically between 10^4^ and 10^6^ TCID_50_/ml) were harvested ten days post-infection and nucleic acid was extracted using an RNeasy Mini Kit (Qiagen, Crawley, UK), according to the manufacturer’s instructions. Optimisation experiments investigated different concentrations of external and internal primer concentrations, the optimum concentration of MgSO_4_ (New England Biolabs, Hitchin, UK), dNTPs and betaine (Betaine 5 M PCR Reagent) (Sigma-Aldrich, Gillingham, UK). An intercalating dye (Picogreen, Molecular Probes, Invitrogen, Paisley, UK) was used to detect amplified DNA and a reference dye (6-ROX, Molecular Probes, Invitrogen, Paisley, UK) was used to monitor any non-specific increases in fluorescence due to temperature fluctuations (as previously described [15,22]). All reactions were performed on an Mx3005p PCR machine (Agilent Technologies, Santa Clara, USA) at an incubation temperature of 65°C for 60 minutes, followed by five minutes at 85°C for inhibition of the *Bst* polymerase (New England Biolabs, Hitchin, UK), and adopted negative control samples that were run in parallel to confirm that non-specific amplification did not occur.

### Loop primers

Following completion of the optimisation process, loop primers were designed for the P32 assay (Table 2). These two primers, known as the FLoop and BLoop, were located between the F1c/B1c and F2/B2 regions respectively (Figure 5) and have been noted to accelerate the detection of target DNA within a LAMP assay [26]. These primers were designed using Primer Explorer V4 software (Fujitsu System Solutions Ltd., Tokyo, Japan).

### Diagnostic sensitivity and specificity

The performance of the P32 LAMP assay was evaluated using a diverse selection of CaPV cell culture isolates (n = 47) from the archived material in the OIE reference laboratory at The Pirbright Institute comprising SPPV (n = 12), GTPV (n = 16) and LSDV (n = 10) strains. Additional CaPV strains that infect both sheep and goats (n = 5) as well as representative vaccine strains (n = 4) were also included in this panel. In order to evaluate the specificity of the LAMP assay, other closely related poxviruses were tested including buffalopox virus, camelpox virus, cheetah poxvirus, cowpox virus, swine poxvirus and Orf virus. Further negative controls (n = 38) included samples collected from cattle skin (n = 16), cattle blood (n = 14) and sheep blood (n = 8) from the United Kingdom where CaPV is not present. For the viral isolates, nucleic acid was extracted using an RNeasy Mini Kit (Qiagen, Crawley, UK), while the blood samples and homogenates prepared from the skin samples were extracted using a BioRobot Universal (Qiagen, Crawley, UK). Previous studies have shown that these extraction protocols generate template DNA from double-stranded DNA viruses that is suitable for amplification by real-time PCR [12,27]. Extracted nucleic acid was stored at −20°C until used. The results generated by the P32 LAMP assay were compared in parallel to a real-time PCR method currently used for the reporting of diagnostic capripox samples [5,12].

### Analytical sensitivity

In order to assess the limit of detection of the LAMP assay, a PCR was used to amplify a large section of the P32 gene, which incorporated the target regions of the LAMP assay. The primer sequences used were 5′- GGA AAT CGT ATG CCG ATG C- 3′ and 5′-TGC TCC CAT TAT AC-3′ which were designed to generate an amplicon of 565 base pairs. The amplified product generated using nucleic acid prepared from the GTPV Vietnam Ninh Tuan 05 isolate was quantified using NanoDrop 1000 Spectrophotometer (Labtech, Ringmer, UK) to determine copy number. This PCR product was then serially diluted to produce a log_10_ dilution series for testing with the CaPV LAMP assay to determine analytical sensitivity of the test.

### Detection of LAMP products using a lateral-flow device

Lateral flow devices (LFD) have previously been successfully adapted for the reporting of nucleic acid amplification by a LAMP assay [22,28]. For application onto the LFD format, the forward internal and backward internal primers used in the P32 LAMP assay were conjugated to Biotin and FITC respectively at the 5′ terminus. Dual labelling with both of these ligands is necessary for detection by a LFD (Forsite Diagnostics, York, UK) which utilises latex beads that bind to one ligand and the membrane line which binds the second ligand. After amplification, the LAMP product was added to the LFD and a blue line was produced at the “test line” which indicated a positive result. Negative samples only generated a blue line at the “control line” that confirmed that the LFD test had been run correctly. In addition to using an LFD, detection of a positive sample was evaluated using a simple colour change reaction as previously described [15]. Following one hour incubation at 65°C, 5 μl of the intercalating dye Picogreen (Molecular Probes, Invitrogen, Paisley, UK) was added directly to either positive or negative samples to show the difference in colour change observed due to the accumulation of amplified DNA.

## Competing interests

The authors confirm that they have no financial or non-financial competing interests.

## Authors’ contributions

LM, LE, KB, ET and VM carried out the experimental work and assisted in the drafting of the manuscript. CO and DK conceived the study and participated in its design and coordination and helped to draft the manuscript. All authors read and approved the final manuscript.
